# Head‐to‐Head Comparison of Plasma vs. Saliva Extracellular Vesicle Subpopulations Derived Normal Controls and Cognitively Impaired Subjects

**DOI:** 10.1002/alz.092263

**Published:** 2025-01-09

**Authors:** Charisse N. Winston, Floyd Sarsoza

**Affiliations:** ^1^ University of Southern California, Alzheimer's Disease Therapeutic Research (ATRI), San Diego, CA USA; ^2^ University of California, San Diego, La Jolla, CA USA; ^3^ Veterans Affairs San Diego Healthcare System, La Jolla, CA USA

## Abstract

**Background:**

The ability to quantify pathogenic proteins related to Alzheimer’s disease (AD) in plasma neuronal extracellular vesicles (NEVs) was vital to their development as diagnostic biomarkers. A similar approach can be employed to assess the biomarker potential of saliva EVs. A head‐to‐head comparison was conducted to characterize plasma and saliva EVs derived patients enrolled in the Nathan Shock Healthy Aging Study, where participants range in age from 30–70+ years, representing the full breadth of the healthy adult human age span.

**Method:**

Matched plasma and saliva EVs were precipitated from two cohorts, normal controls (NC, 55+ years of age) and patients with mild cognitive impairment (MCI; 55+ years of age) as defined by the Modified Mini‐Mental State Test (3MS). EVs were precipitated and enriched against neuronal‐origin, L1CAM using magnetic immunocapture and fluorescence‐activated cell sorting. EVs and NEVs were characterized for size and shape using NanoSight and marker profiling using Western Blot. AD‐related pathogenic proteins Aβ40–42, p‐tau, and Nf‐L will be quantified in EVs using SIMOA assays.

**Result:**

Plasma and saliva EVs derived from NC and MCI subjects demonstrated similar size distributions and shape characteristics as previously reported. EV marker profiling revealed differential expression of well characterized EV and cellular markers in saliva and plasma EVs. We determined that the rabbit monoclonal antibody raised against tetraspannin CD81 (Abcam) did not identify populations of plasma and saliva EVs while mouse monoclonal antibody against CD81 (Santa Cruz) identified separate populations of plasma and saliva EVs. CD9, TSG101 and ALIX markers positively identified subpopulations of EVs in plasma and saliva while CD63+ EVs were only observed in plasma EVs derived from NC and MCI subjects. MAP2 confirmed neuronal origin of NEVs derived from the plasma of NC and MCI subjects.

**Conclusion:**

The presence of different subpopulations of EVs in plasma and saliva may impact their AD biomarker potential and requires further investigation. Additional analyses will be employed to confirm neuronal origin of EVs from saliva. EV cargo analyses will be performed to assess the biomarker potential of plasma and saliva EVs derived from NC and MCI subjects.

